# Machine Learning-Based Optimization of Mechanical and Morphological Performance of Polylactic Acid Nanocomposites with Lignin Nanoparticles

**DOI:** 10.3390/polym18151811

**Published:** 2026-07-24

**Authors:** Erol Imren, Deniz Aydemir, Anton Kuzmin, Sezgin Koray Gülsoy, Ömer Ümit Yalçın, Yasemin Şimşek Türker, Petr Pantyukhov

**Affiliations:** 1 Department of Forest Industrial Engineering, Faculty of Forestry, Bartin University, 74100 Bartin, Türkiye; denizaydemir@bartin.edu.tr (D.A.); sgulsoy@bartin.edu.tr (S.K.G.); 2 Department of Mechanization of Agricultural Products Processing, National Research Mordovian State University, Saransk 430005, Russia; kuzmin.a.m@yandex.ru; 3 Scientific Laboratory of Advanced Composite Materials and Technologies, Plekhanov Russian University of Economics, Moscow 117997, Russia; 4 Department of Forest Industrial Engineering, Faculty of Forestry, Isparta University of Applied Sciences, 32040 Isparta, Türkiye; omeryalcin@isparta.edu.tr; 5 Department of Civil Engineering, Faculty of Engineering and Natural Sciences, Suleyman Demirel University, 32040 Isparta, Türkiye; 6 Emanuel Institute of Biochemical Physics, Russian Academy of Sciences, Moscow 119334, Russia; pantyukhov@mail.ru; 7 Higher Engineering School of New Materials and Technologies, Plekhanov Russian University of Economics, Moscow 117997, Russia

**Keywords:** polylactic acid (PLA), lignin nanoparticles, maleic anhydride compatibilization, machine learning optimization, biopolymer nanocomposites

## Abstract

This study investigates machine learning-based optimization of the mechanical properties of environmentally friendly biopolymer nanocomposites produced by incorporating lignin nanoparticles (NLPs) and maleic anhydride (MA) into a polylactic acid (PLA) matrix. Lignin was extracted from black pine using a deep eutectic solvent method and melt-compounded with PLA via twin-screw extrusion, followed by injection molding. Mechanical performance was evaluated using tensile and three-point bending tests, while fracture morphology was examined by scanning electron microscopy (SEM). Random Forest (RF) and Extreme Gradient Boosting (XGBoost) models were applied to predict and optimize mechanical properties using lignin and MA contents as input variables, with an 80/20 training–testing data split. Experimental results showed that neat PLA exhibited the highest tensile strength (61 MPa) and modulus (5.2 GPa). The addition of low lignin contents with MA slightly reduced tensile properties but significantly enhanced flexural strength (≈58 MPa) and modulus (≈3.9 GPa). SEM observations revealed uniform nanoparticle dispersion and crack-free fracture surfaces at low lignin loadings, whereas higher lignin contents resulted in agglomeration and brittle behavior. Both machine learning models demonstrated high predictive accuracy, with RF outperforming XGBoost. The results confirm that MA improves interfacial adhesion and that data-driven approaches effectively support optimization of biopolymer nanocomposite compositions.

## 1. Introduction

Synthetic plastic production has increased recently, reaching about 350 Mt in 2022. If the current trend continues, global plastic consumption is predicted to increase from 464 Mt in 2020 to about 884 Mt by 2050. Moreover, the rapid rise in fossil fuel consumption has increased plastic pollution and global warming [1,2,3,4,5]. Many researchers have been using lignocellulosic fillers to enhance the biodegradability of conventional plastics, thereby reducing global pollution and mitigating the environmental impact [6,7,8]. Lignocellulosic materials, including lignin and cellulose, offer a renewable route to reduce the dependence on finite petrochemical feedstocks [9,10]. Lignin, the world’s second most abundant renewable polymer, is mainly known as a low-value waste in the pulp-and-paper industry. When blended with biopolymers, such as poly (lactic acid) (PLA) or polyhydroxybutyrate (PHB), it can be formed into a fully “green” high-performance composite [11,12]. It can be efficiently isolated from lignocellulosic biomass using DESs, which are emerging as green and sustainable alternatives to chemicals used in traditional pulp production [13,14]. DESs are obtained by mixing a hydrogen bond donor and acceptor, yielding a biodegradable, non-toxic, and easy-to-prepare solvent system. DES-based lignin extraction offers superior properties compared to traditional methods, including milder processing conditions and reduced environmental impact. Lignin extracted with DESs can be readily used in high-value applications such as bioplastics [15], adhesives [16], and biomedical materials [17].

PLA is another biopolymer known for its processability, non-toxicity, biodegradability, and biocompatibility. However, it suffers from limited tensile strength, low elongation at break, low thermal resistance, and slow crystallization [12,18]. Despite this, lignocellulosic fillers, including lignin and cellulose, exhibit good mechanical performance and natural biodegradability [19,20]. Many studies have examined their incorporation into PLA to improve mechanical strength, thermal stability, and crystallinity. For example, Triwulandari et al. showed that lignin increased the biodegradation rate of PP/PLA blends but slightly decreased their mechanical strength [21]. Lignin fillers obtained from walnut-shell biomass with DESs were reported to decrease mechanical strength values by restricting the melt mobility and raising the viscoelastic response of PLA [22]. At the same time, Kraft lignin improved mechanical strength values, antioxidant activity, and thermal stability in the 300–350 °C range [23]. Surface-modified lignin (COOH lignin) was found to slightly enhance the interfacial adhesion between lignin and PLA [24,25]. All of these studies on lignin–PLA blends have shown that the interfacial interaction between lignin and the PLA matrix does not improve significantly. In addition, increasing brittleness imposes major limitations on mechanical performance. These findings suggest that, beyond blending the two components, additional surface-active agents or compatibilizers are essential for their application. As a result, compatibilizers such as silanes and maleic anhydride are increasingly employed in similar scientific studies. MA, one of the most common compatibilizers, is widely used to improve the interaction of lignocellulosic fillers. In a similar study, PLA was grafted onto lignocellulosic hydroxyl groups, improving fiber–matrix bonding and mechanical, thermal, and morphological properties [26,27]. Ye et al. demonstrated that tailored MA and lignin loadings synergistically enhanced the performance of PLA composites [28]. In the present study, PLA/lignin nanocomposites were produced and systematically examined to determine how varying lignin and MA contents affect their morphological and mechanical behavior. Makri et al. produced PLA composites with macro lignin and nano lignin synthesized via in situ reactive processing, and the results were similar to those reported previously [29]. Generally, adding lignin was found to positively affect the PLA–lignin interaction and properties. Recent studies have demonstrated that the molecular structure of lignin plays an important role in determining the material properties of polymer composites with lignin. The distribution of phenolic hydroxyl, aliphatic hydroxyl, methoxy, and aromatic groups in the lignin structure provides intermolecular interactions with polymer matrices via hydrogen bonding, hydrophobic interactions, and π–π stacking, thereby influencing lignin dispersion, interfacial adhesion, and, ultimately, the mechanical performance of composites with lignin. Also, its molecular weight, structural heterogeneity, and chemical modifications improve degradation behavior by altering polymer compatibility, water accessibility, and hydrolytic degradation pathways. Therefore, tailoring lignin chemistry is considered an effective strategy for simultaneously optimizing composite performance and degradation characteristics [30,31].

Recent nanoengineering strategies have further improved dispersion. Ethyl acetate self-assembled lignin nanospheres with sizes ranging from 200 to 400 nm were used. Even at a low loading level of 0.5 wt%, these nanospheres reduced melt viscosity. In fused-deposition-modeled parts, flexural strength increased by 131%, tensile strength by 56%, and impact strength by 14%. [32]. Despite these benefits, adding lignin (>5–10 wt%) at high loading levels often led to aggregation due to a polarity mismatch with PLA, thereby increasing crystallinity and elongation at break [33]. As shown in the compatibilization routes, the plasticizers, including PEG and epoxidized palm oil, and the reactive coupling agents, including glycidyl ethers and maleic anhydride, were found to be indispensable for restoring interfacial adhesion and toughness [34,35,36]. Although the chemical modification of lignin and the matrix provided functionality for PLA–lignin blends, increased processing costs and ecological concerns necessitated data-driven formulation screening. Machine learning (ML) methods have now provided effective approaches to the classical design of experiments by p redicting property–composition relationships with far fewer trials. With a training dataset of only 13 experimental points, Multilayer Perceptron (MLP) networks successfully captured the nonlinear interactions between PLA, lignin, and epoxidized palm oil—achieving for tensile strength to guide optimal formulation [37]. Broader reviews have shown that supervised and deep learning algorithms accelerate composite discovery, enabling virtual screening of thousands of formulations and real-time process optimization for greener manufacturing [38]. Recently, machine learning has emerged as a powerful tool for reducing experimental effort and accelerating the design of sustainable composite materials. By establishing predictive relationships between processing parameters and material performance, machine learning enables efficient optimization while minimizing the number of experimental trials. For example, Saha et al. [39] successfully employed XGBoost and Random Forest algorithms to predict the impact strength and cost of hybrid polymer composites, demonstrating the superior predictive capability of the XGBoost model for composite optimization. In conventional experimental approaches, optimization of polymer composite formulations typically relies on extensive trial-and-error procedures, which are both time-consuming and resource-intensive. In contrast, ML techniques can capture complex, nonlinear relationships between composition variables and material properties, making them particularly suitable for multicomponent systems such as PLA/lignin/MA composites. Despite these advantages, the application of ML in biopolymer nanocomposites is still limited, especially in studies combining environmentally friendly lignin sources with compatibilized systems. Moreover, there is a lack of comparative studies evaluating ensemble learning algorithms, such as Random Forest and XGBoost, for predicting and optimizing mechanical performance using relatively small experimental datasets. Therefore, this study aims to address this gap by employing data-driven modeling to optimize composite formulations while efficiently minimizing experimental effort. Although artificial neural networks (ANNs) have been successfully applied for predicting the properties of polymer composites, their performance generally depends on the availability of sufficiently large datasets and careful hyperparameter optimization. In the present study, the experimental dataset consisted of only 20 formulations. Therefore, ensemble learning algorithms, namely Random Forest and XGBoost, were preferred because they generally provide more stable and robust performance on relatively small structured datasets while effectively capturing nonlinear relationships between composition and mechanical properties.

Although PLA–lignin blends have been investigated in prior studies, optimizing mechanical and morphological properties using machine learning remains unexplored. Most previous studies focused on lignin nanoparticles produced via chemical processes, whereas this study used a green production method. The present study aimed to optimize the mechanical performance of PLA/lignin nanocomposites by integrating controlled melt-compounding with Random Forest-assisted predictive modeling, thereby identifying the minimum lignin and maleic anhydride loadings that maximize strength, toughness, and interfacial homogeneity while preserving biodegradability.

## 2. Materials and Methods

### 2.1. Materials

In this study, PLA was supplied from Zhejiang Hisun Biomaterials Co. (Taizhou City, China). The material properties for PLA were tensile strength and modulus of 35 MPa and 3.3 GPa, elongation at break of 4.0%, Izod impact strength of 36 J·m^−1^, density of 1.24 g/cm^3^, and melt flow index of 10±3 g/10 min. Black Pine (*Pinus nigra* Arn.) wood used for lignin production was purchased from Donmezler Co. (Bartin, Türkiye). Choline chloride (CAS No. 67-48-1) and lactic acid (CAS No. 79-33-4), used as components of the deep eutectic solvent (DES), were obtained from Sigma-Aldrich (Istanbul, Türkiye). Maleic anhydride (MA), supplied by Sigma-Aldrich as a modifier, was used to improve the interaction between the polymer and lignin fillers.

### 2.2. The Production of Lignin with an Environmentally Friendly Approach by Using Deep Eutectic Solvents

**Figure 1:**
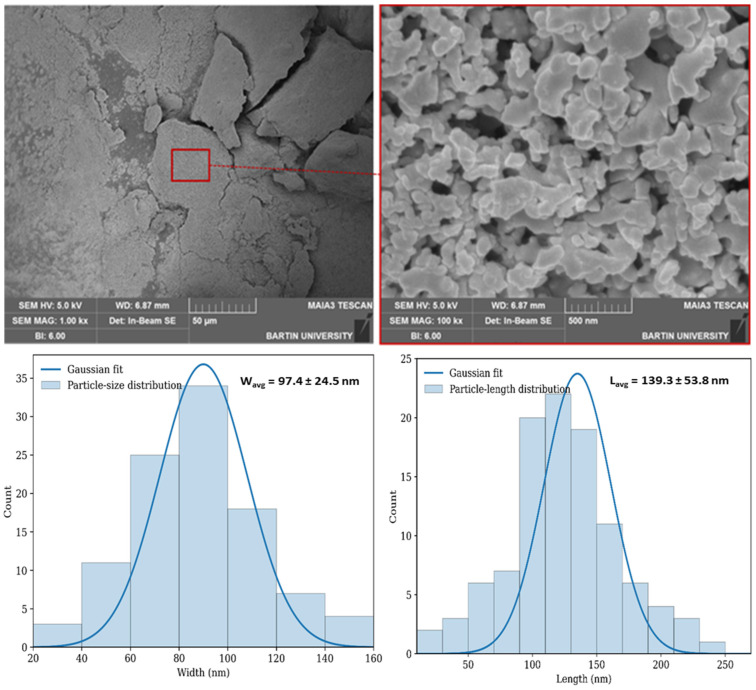
Particle size distribution of the NLPs.

In this study, a DES composed of choline chloride and lactic acid (1:9 molar ratio) was prepared via heating at 80 °C for 60 min until a clear, homogeneous solution was obtained, as defined by Gulsoy et al. [40]. The DES was stored in a vacuum desiccator until use. A total of 700 g of European black pine (*Pinus nigra* Arn.) wood chips was pulped in a 15 L lab-scale rotary digester using 3500 g of the DES (a 1:5 wood-to-DES ratio) at 160 °C for 4 h. Following cooking, the black liquor and pulp were separated by filtration. For lignin precipitation, 5 times the volume of distilled water was added to the black liquor. Water served as an anti-solvent, facilitating lignin precipitation by disrupting hydrogen bonding between the DES and lignin. The precipitated lignin was collected on Whatman no. 42 filter paper, oven-dried at 30 °C for 24 h, and ground in a Wiley mill. Moisture content was determined to be 6%. The obtained NLPs were stored in a plastic bag in a climatic room at 20 °C and 60% relative humidity before the preparation of the biopolymer-based biocomposites. Particle size distribution of the NLPs is given in Figure 1.

Particle size distribution analyses (width and length) of the NLPs were performed using microscopic image analysis. The acquired images were processed and calibrated using Adobe Photoshop, and the obtained dimensional data were plotted using OriginPro 2026b software, academic version.

### 2.3. Preparation of Biopolymer-Based Biocomposites with Lignin Nanomaterials

The PLA blends, including PLA, MA, and lignin nanoparticles, were prepared using a HAAKE Rheomex OS PTW16 co-rotating twin-screw extruder (Haake Technik GmbH, Vreden, Germany) with an L/D ratio of 40:1, as presented in Table 1. The MA loading ratio was changed from 3 to 10 wt.% to examine its effectiveness as a compatibilizing agent. A low MA loading ratio may provide sufficient interfacial interaction between lignin and the matrix, whereas a high MA loading ratio may cause the deterioration of the composite morphological structure. To determine the optimum MA level, the various concentration range was selected. The extruder has 10 heated zones. The temperature in each zone was 180 °C, and the screw rotation speed was 150 rpm. After extrusion, the blends were cooled in a water bath and then ground into pellets. The pellets (granules) were subsequently dried at 40 °C for 24 h.

The dried pellets were used to obtain mechanical test samples on a Cronoplast injection molding machine (Cronoplast Sl, Barcelona, Spain) at a mold temperature of 30 °C, a plasticizer temperature of 180 °C, an injection cylinder temperature of 185–190 °C, and an injection pressure of 56 MPa. The obtained test samples were stored for 2 days in a climatic chamber at 23 °C and 65% RH.

### 2.4. Methods

#### 2.4.1. Mechanical Properties

The mechanical performance of the samples was assessed through tensile and flexural tests. Before testing, all specimens were conditioned by drying at 40 °C for 24 h to ensure uniformity. Each test was conducted with at least 6 replicates, and the average values are reported in accordance with the corresponding ASTM standards. Tensile properties were measured using a Utest mechanical testing machine (Utest, Ankara, Türkiye) on specimens sized 115 × 13 × 3 mm, at a crosshead speed of 5 mm/min, following ASTM D638 Type I [41]. Tensile strength and modulus were calculated according to the formula below:

$$Tensile\;strength\;(TS)=\;\frac{F}{A}\;\frac{N}{{mm}^{2}}\;Tensile\;modulus\;\left(TM\right)=\;\frac{Stress\;(\sigma )}{Strain\;(\varepsilon )}\;=\;\frac{\frac{F}{A}}{\frac{∆L}{L_{o}}}=\;\frac{F\;\times \;L_{o}}{A\;\times \;∆L}\;\frac{N}{{mm}^{2}}$$

where *F*, *A*, Δ*L*, and *L*_0_ represent the maximum force at fracture, the original cross-sectional area, the change in length (extension), and the original gauge length before tensile loading, respectively. In the calculation of tensile modulus, stress and strain data from the linear or elastic region between 0.01% and 0.05% strain were used.

Flexural strength was evaluated using a three-point bending setup with samples measuring 80 × 12 × 3 mm at a loading rate of 2 mm/min, according to ASTM D790 [42]. The elongation at break was calculated from the flexural test data. Flexural strength and modulus were calculated according to the formula below;

$$Flexure\;strength\;(FS)\;=\frac{3\times F\times L}{2\times w\times h^{2}}\;\frac{N}{{mm}^{2}}\;Flexure\;modulus\;\left(TM\right)=\frac{L^{3}\times F}{4\times w\times h^{3}\times d}\;\frac{N}{{mm}^{2}}$$

where *L*, *w*, *h*, and *d* represent the support span, specimen width, specimen thickness (depth), and mid-span deflection, respectively.

#### 2.4.2. Scanning Electron Microscopy (SEM)

Morphological characterization of the samples was carried out using a Tescan MAIA3 XMU scanning electron microscope (Brno, Czechia). Before imaging, the sample surfaces were sputter-coated with a palladium/gold alloy to improve electron conductivity and image resolution. The acceleration voltage for the settled electron was generally set to about 5 kV, depending on the sample’s surface conductivity.

#### 2.4.3. Machine Learning Methods Used for Predictive Purposes

Extreme Gradient Boosting (XGBoost) Algorithm:

The XGBoost algorithm, developed by Chen and Guestrin [43], builds upon the Gradient Boosted Decision Trees (GBDTs) framework version 3.1.0. Its outstanding performance in various machine learning competitions, particularly on the Kaggle platform, has attracted significant interest [44].

(1) $$O=\sum_{i=1}^{n}L(y_{i},F\left(x_{i}\right))+\sum_{k=1}^{t}R\left(f_{k}\right)+C$$

where C is a constant that may be selectively eliminated, and $R\left(f_{k}\right)$ is the regularization term at iteration k.

Written as the regularization term $R\left(f_{k}\right)$:

(2) $$R\left(f_{k}\right)=aH+\frac{1}{2}\eta \sum_{j=1}^{H}w_{j}^{2}$$

Here, H is the number of leaves, η is the penalty variable, and $w_{j}$ is the output value for each leaf node. α is the leaf complexity. While a leaf node represents a tree node that cannot be split, a leaf represents the predicted category based on classification criteria. Furthermore, unlike GBDTs, which rely on first-order derivatives, XGBoost uses a second-order Taylor expansion of the objective function. When the mean squared error (MSE) is employed as the loss function, the main function can be formulated as follows:

(3) $$O=\sum_{i=1}^{n}[p_{i}w_{q(x_{i\;})}+\frac{1}{2}(q_{i}w_{q\left(x_{i\;}\right)}^{2})]+aH+\frac{1}{2}\eta \sum_{j=1}^{H}w_{j}^{2}$$

where $q_{i}$ and $h_{i}$ stand for the loss function’s representation, and $q(x_{i})$ is a function that maps data points to leaves. The sum of the individual loss values yields the final loss value. By incorporating first- and second-order derivatives, the overall objective loss is computed as the sum of the individual losses across each leaf node. Since the loss values of the leaf nodes are aggregated, the primary function can be represented accordingly. In this context, the samples in the decision tree (DT) are associated with the leaf nodes, and the total loss is calculated by summing the losses at these nodes. Therefore, the main function can be formulated as follows:

(4) $$O=\sum_{j=1}^{T}[p_{j}w_{j}+\frac{1}{2}(Q_{j}+\eta w_{j}^{2})]+aH$$

where $P_{j}=\sum_{i\in I}p_{i},\;Q_{j}=$, and $I_{j}$ is the total number of samples in leaf node j.

In conclusion, optimizing the primary function becomes easier by identifying the minimum of the quadratic function, which introduces the concept of regularization. This regularization helps XGBoost to prevent overfitting more effectively. Due to its inherent structure, XGBoost is particularly efficient at mitigating overfitting.

Random Forest (RF) Algorithm:

Random Forest (RF) is one of the most popular machine learning techniques due to its flexibility and ease of use. Developed by Breiman [45], this supervised learning method is used for both regression and classification tasks. RF is an ensemble learning method that aggregates information from multiple decision trees (DTs) and, depending on the task, uses either the mean of the results or majority voting to improve prediction accuracy. For instance, given an input dataset $Q\;=\;\{q_{1},\;q_{2},\;q_{3},\;\ldots ,\;q_{n}\}$, where n represents the total number of data points, the RF model is composed of a collection of T trees, such as $T_{1}(Q)$, $T_{2}(Q)$, $T_{3}(Q),\;\ldots ,\;T_{n}(Q)$. The predictions from these trees are $R_{1}$, $R_{2},\;\ldots ,\;R\text{\_}n$. In regression problems, the final prediction is the average of all trees’ results. Decision tree algorithms work by splitting the initial training set into smaller subsets, with only a few randomly selected predictor variables considered at each split. Decision trees can grow without limits unless manually pruned according to predefined stopping criteria. Common stopping criteria include metrics like RMSE, MSE, and the Gini Diversity Index. In the RF model, trees that make accurate predictions are retained, while those that make inaccurate predictions are discarded. This random selection of decision trees and predictor variables helps mitigate overfitting, a common issue in single-decision-tree models [45,46].

Implementation and Evaluation Methods of ML Models:

In this study, machine learning models are constructed using Jupiter Lab 3.0.14 and written in Python 3.14.6. The three main packages are xgboost 1.6.2, tensorflow 2.9.1, and sklearn 1.0.2. Three measures were used to assess the model’s predictive accuracy: R^2^, Mean Absolute Error (MAE), and Root Mean Square Error (RMSE). The degree of model fit is indicated by $R^{2}$. The residuals’ variance is measured using RMSE. The MAE calculates the absolute difference between the projections and observations. The following equations are displayed:

(5) $$R^{2}=\frac{{[\int_{i=1}^{n}\left(f_{i}-‵f\right)-(o_{i}-‵o)]}^{2}}{[\int_{i=1}^{n}{\left(f_{i}-‵f\right)}^{2}]-[\int_{i=1}^{n}{\left(o_{i}-‵o\right)}^{2}]}$$

(6) $$RMSE=\sqrt{\frac{1}{n}\sum_{i=1}^{n}{\left(f_{i}-o_{i}\right)}^{2}}$$

(7) $$MAE=\frac{1}{n}\sum_{i=1}^{n}|f_{i}-o_{i}|$$

where, for all i cases up to the n instances, f denotes forecast and o denotes observation. The models are intended for interpolation within the studied compositional domain, and extrapolation beyond this range may lead to reduced prediction reliability.

The predictive performance reported in this study was obtained using a single random 80/20 train–test split. Although k-fold cross-validation generally provides a more robust estimate of model generalization, the limited dataset size and the objective of evaluating interpolation performance within the investigated compositional domain motivated the use of a single train–test partition. Future studies with larger datasets will employ k-fold cross-validation to validate model robustness further. The Random Forest and XGBoost models were implemented using the standard implementations available in the Scikit-learn (v1.3.x) and XGBoost Python libraries (v2.0.x). The models were trained under identical conditions to enable a fair comparison between the two algorithms.

## 3. Results and Discussion

### 3.1. Mechanical Properties

**Figure 2:**
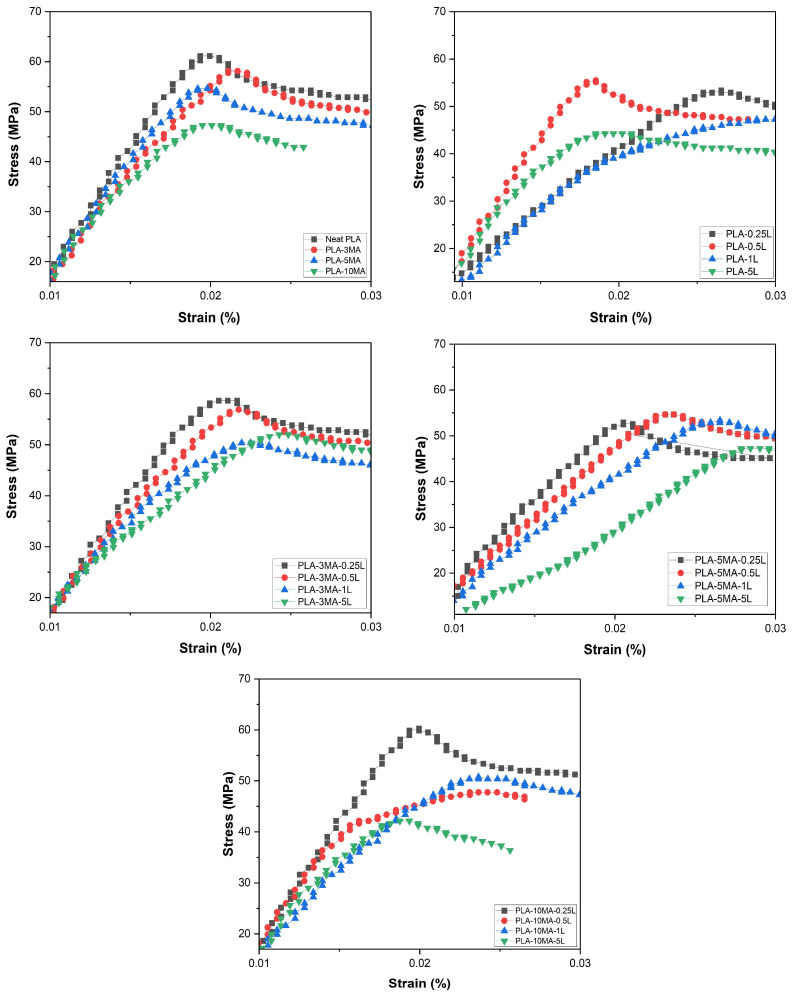
Stress–strain curves of the lignin-reinforced PLA composites in the tensile test.

**Figure 3:**
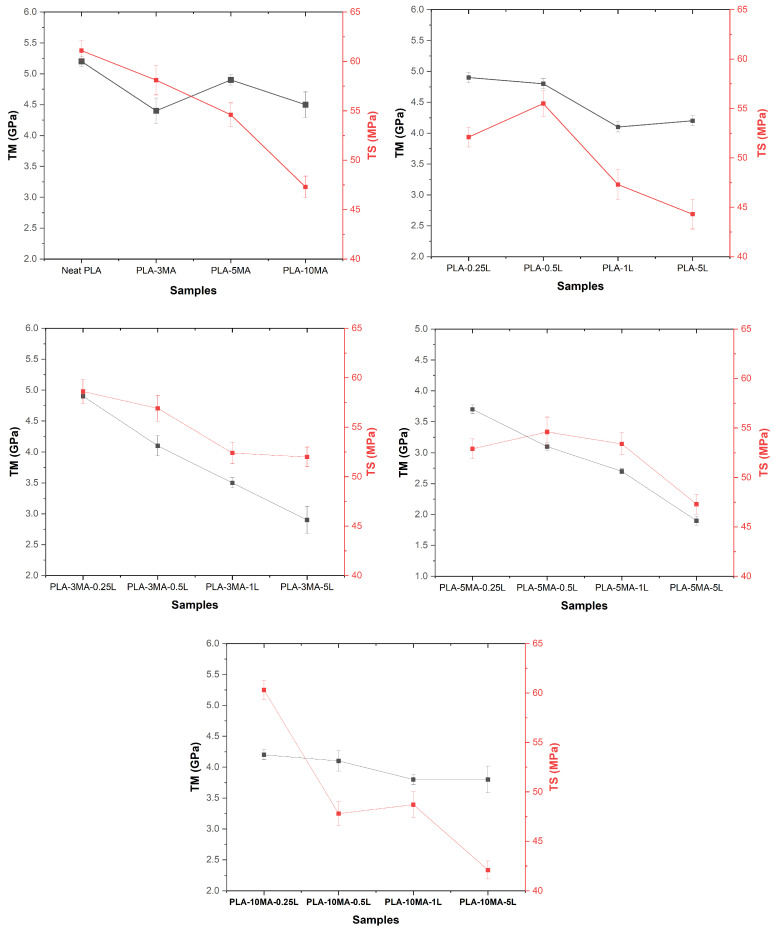
Tensile strength and modulus of the lignin-reinforced PLA composites.

The mechanical properties of polymers and polymer composites are essential for determining their response to applied stresses, which directly influence their suitability for various applications and expected service life. Therefore, tensile and flexural tests for the polymer nanocomposite blends were conducted according to the relevant standard, and the tensile stress–strain curves and tensile strength and modulus are shown in Figure 2 and Figure 3, respectively. A one-way analysis of variance (ANOVA) was performed at the 95% confidence level to determine whether statistically significant differences existed among the mechanical results of the composite groups. Since significant differences were detected among the results, Duncan’s multiple range test was applied to identify the variations between groups. In Table 2, statistically significant differences among the mechanical properties including TS, TM, FS, FM, and EatB of the composites are indicated by different letters, such as A, B, and C. The Duncan test showed distinct ranking patterns among the composites, showing that the combined effects of MA and lignin content more strongly influenced the mechanical response than either factor alone.

According to Figure 2, the maximum stress and strain reached about 60 MPa and 5% for neat PLA, and the addition of MA to neat PLA decreased the stress and strain values. Adding MA (3%) and lignin (0.25%) at a low loading ratio resulted in better stress and strain, whereas MA (10%) and lignin (5%) at a high loading ratio yielded the lowest mechanical properties due to limited interaction between the polymer matrix and lignin.

The addition of MA and lignin can be seen to affect the tensile properties of PLA, as shown in Figure 2. The addition of MA to neat PLA decreased the tensile strength, and while the MA loading ratio increased from 3% to 10%, and the loss of TS decreased further. The lowest TS was 47.3 MPa (29% loss) for PLA-10MA. Similarly, the lowest tensile modulus was determined to be 4.5 GPa (14% loss) for PLA-10 MA. From here, it can be said that the addition of MA decreased both tensile strength and modulus. The reduction is also due to structural irregularities and localized stress concentrations within the PLA-MA blends, which weaken the overall mechanical performance, as reported by Chauhan et al. [47]. The addition of lignin to neat PLA generally had similar effects on TS and TM, except for PLA-0.5L, and the lowest TS and TM were 44.3 MPa (15% loss) and 4.1 GPa (20% loss), respectively, for PLA-5L and PLA-1L. This status has been attributed in previous studies to the poor interfacial compatibility between PLA and lignin, which limits effective stress transfer within the composites, as found by Ye et al. [33].

**Figure 4:**
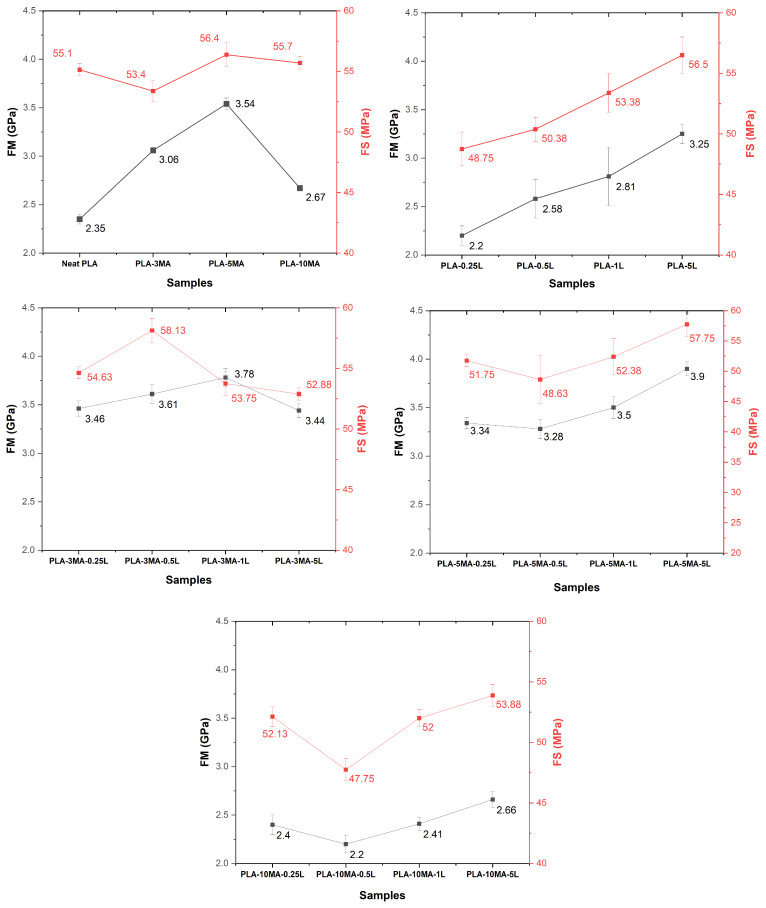
Flexural strength and modulus of lignin-reinforced PLA composites.

**Figure 5:**
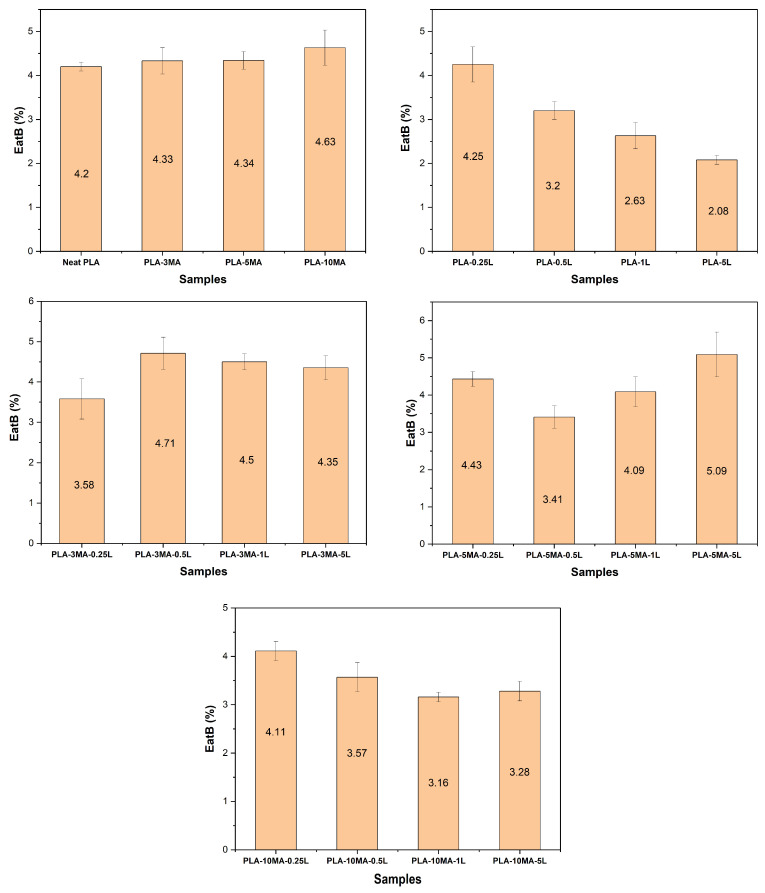
Elongation at break of samples.

In the PLA composite with both MA and lignin, it was observed that both TS and TM decreased as the content of MA and lignin was raised in the PLA-3MA-L blends. The lowest TS and TM values were 52 MPa (11% loss) and 2.9 GPa (41% loss), respectively, for PLA-3MA-5L. Similarly, in PLA-5MA-L composites, although the TM continued to decrease, a slight increase in TS was observed for PLA-5MA-0.5L, reaching 54.6 MPa. However, the lowest TM was determined to be as low as 1.9 GPa (49%) for PLA-5MA-5L. For PLA-10MA-L composites, a similar trend was observed: increasing the MA and lignin contents reduced TS and TM, with the lowest TS and TM values of 42.1 MPa (30.2% loss) and 3.8 GPa (10%), respectively, for PLA-10MA-5L. As a result, it was concluded that the addition of either MA or lignin to PLA composites did not improve tensile strength or modulus. This behavior is consistent with previous studies, which have attributed such decreases to insufficient interactions between MA and PLA, as well as the limited interfacial compatibility between the polar lignin and the relatively non-polar PLA matrix. The flexural strength and modulus of the lignin-reinforced PLA composites are shown in Figure 4, and the elongation at break of the samples is shown in Figure 5.

As shown in Figure 4, the FS and FM values of neat PLA were clearly affected by the incorporation of varying amounts of MA and lignin. The addition of MA at varying ratios did not cause a significant change in FS values, with the lowest and highest FS values observed as 53.4 MPa (5% decrease) for PLA-3MA and 56.4 MPa (3% increase) for PLA-5MA. In contrast, FM values increased with the addition of MA up to 5%, reaching 3.54 GPa (51%), and then decreased to 2.67 GPa (12%) at 10% MA. These results suggest that increasing MA from 3% to 5% can enhance PLA stiffness, whereas higher concentrations (e.g., 10% MA) may disrupt matrix homogeneity and reduce rigidity [47]. In PLA samples containing lignin, a consistent increase in both FS and FM was observed with increasing lignin content. The highest FS and FM values were found in PLA-5L samples as 56.5 MPa (14% increase) and 3.25 GPa (32% increase), respectively. This enhancement indicates sufficient interaction between lignin and the polymer matrix, especially in facilitating stress transfer during bending, thereby improving both stiffness and fracture resistance. In PLA composites containing both MA and lignin, FS and FM generally increased with rising MA and lignin contents. In composites with 3% MA, FS increased to 58.13 MPa (6% increase) with lignin addition, but subsequently decreased to 52.88 MPa (3% loss). Similarly, FM increased up to 3.78 GPa (9% increase) at 1% lignin content, but declined to 3.44 GPa (1% loss) at 5% lignin. For composites with 5% MA, although FS and FM initially decreased with the addition of 0.25% lignin, they increased at higher lignin contents, reaching 57.8 MPa (12% increase) and 3.9 GPa (17% increase), respectively, at 5% lignin. A similar decrease–increase trend was observed for 10% MA composites, with the highest FS and FM values of 53.88 MPa (4% increase) and 2.66 GPa (11% increase), respectively.

As a result, the highest FS value of 58.13 MPa was obtained in the PLA-3MA-0.5L sample, while the highest FM value of 3.9 GPa was observed in the PLA-5MA-5L sample. These results indicate that the combined use of MA and lignin at low-to-moderate levels may exert a synergistic effect on the mechanical properties of PLA composites. In summary, the combination of low concentrations of MA and lignin improved both FS and FM, whereas higher concentrations were less effective. According to the EatB results, the addition of MA to neat PLA slightly improved ductility, particularly up to 5% MA content, whereas a further increase to 10% MA had a negative impact on EatB. When lignin was added alone, a significant decrease in EatB was observed, indicating that lignin contributes to stress concentration within the matrix, thereby increasing brittleness. However, when MA and lignin were used together, especially at 3–5% MA and 0.5–1% lignin levels, a balanced, positive effect on ductility was observed. The highest EatB value was 5.1% for the PLA-5MA-5L sample, while the lowest was 2.1% for the PLA-5L sample. These findings suggest that MA enhances the interfacial compatibility between lignin and PLA, helping to maintain ductility even at high lignin contents. Nevertheless, it was determined that higher MA levels (>10%) may induce phase separation in some formulations, thereby limiting this positive effect.

The different trends observed in tensile and flexural strengths with adding lignin are attributable to the different stress states and failure mechanisms occurring under these two test methods in the brittle materials presented by Leguillon et al. [48]. In tensile testing, the entire cross-section of the PLA/lignin composites is subjected to uniform uniaxial tensile stress, and lignin particles often act as stress concentrators due to poor interfacial adhesion and agglomeration. Under tension loading, these weak interfaces readily initiate microcracks that propagate rapidly, leading to premature failure and reduced tensile strength. In flexural testing, the specimen is subjected to a combination of tensile, compressive, and shear stresses, with the maximum stresses concentrated primarily on the outer surfaces of the PLA/lignin composites, and lignin particles, being rigid and highly stiff, can effectively resist the compressive stresses acting on the surfaces of the composites during flexure. For this reason, the results of bending and tensile strengths usually do not show much similarity.

### 3.2. Morphological Properties

**Figure 6:**
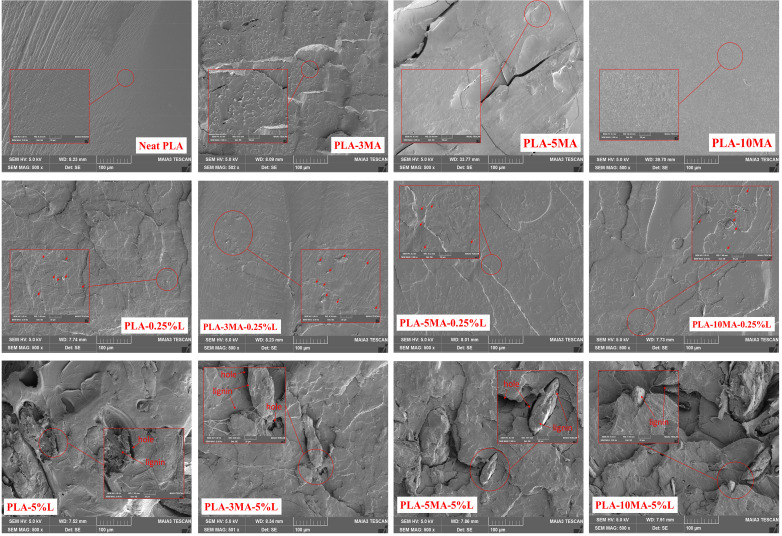
SEM images of PLA-based biocomposites.

SEM investigations were conducted on the various fracture surfaces of the samples under tensile testing, as seen in Figure 6. The samples with MA content of 3, 5, and 10% had a brittle-type fracture, and the surfaces on the samples with 3% MA had various micron-sized cracks; also, the samples with 5% MA had macro-sized and large cracks. However, the samples with 10% MA showed a smooth fracture surface. Compared with neat PLA, the samples containing 0.25% MA-grafted lignin exhibited a smoother fracture surface without visible cracks. The micron-sized lignin particles were detected on the fractured surfaces, and the low-content particles were observed to be more homogeneously dispersed. No crack formation was detected on the fractured surfaces of the samples with MA-0.25%L, indicating better mechanical performance, as seen in Figure 3 and Figure 4. In the samples with MA-5% L, particle dispersion was nonhomogeneous, leading to the formation of various cracks and voids around lignin particles. In the samples with high lignin content (3 and 5%), voids and cavities with diameters ranging from 15 to 50 µm were generally observed at the interfaces between the lignin aggregates and the matrix, as seen in Figure 5. The aggregation of the lignin particles may have caused a possible particle-induced stress concentration. As the percentage of lignin was raised from 0.25% to 5%, the tendency of lignin particles to aggregate became visible, indicating a lower mechanical performance, as shown in Figure 2 and Figure 3. This behavior led to a more brittle fracture in the samples containing 0.25% lignin. In contrast, samples with 3% and 5% lignin exhibited fracture characterized by a combination of brittle failure and increased plastic deformation. This phenomenon is particularly evident from the high density of voids observed at the interfaces between lignin aggregates and the polymer matrix.

Furthermore, detailed examination of the fracture surfaces indicates insufficient interfacial adhesion between the matrix and lignin particles, suggesting ineffective stress transfer across the interface. In contrast, the use of 3% MA resulted in improved interaction between lignin and PLA. In contrast, at 5% MA, this interaction deteriorated, leading to the formation of larger voids between lignin and PLA and a reduction in interfacial adhesion. These findings indicate that 3% MA is sufficient for achieving optimal compatibility.

A study by Birnin-Yauri et al. [27] found that incorporating 3% MA improved the interfacial interaction between lignin and PLA, attributed to the formation of chemical interactions between MA-grafted PLA and lignin hydroxyl groups. However, at higher MA content (5%), this interaction deteriorated, leading to increased void formation and reduced interfacial adhesion [39,40]. Similar observations have been reported, in which excessive compatibilizer content led to phase separation or ineffective compatibilization. Additionally, poor compatibility between lignin and PLA can lead to interfacial voids and reduced stress transfer [24,49]. Therefore, in this study, it can be concluded that 3% MA was an optimal compatibilizer content.

In a similar study, the same type of particle stacking, along with holes and voids, was found to be responsible for the lower mechanical performance of polymer composites with lignin [28,29]. In another study, it was found that the addition of lignin particles led to the formation of air bubbles in the polymer composites, which nucleated cracks and holes during compounding, thereby reducing mechanical properties [12,17,23].

As a result, the fractured surfaces of the samples with MA-5 wt% L exhibited a heterogeneous and brittle morphology, characterized by irregularly distributed voids and noticeable particle agglomerations. In particular, the surface texture of these samples appeared rough, with distinct microcavities likely caused by phase separation or insufficient interfacial adhesion between the lignin particles and the PLA matrix. Moreover, varying the MA content did not result in a significant improvement in morphological characteristics. SEM images consistently revealed localized lignin agglomerates, attributed to poor lignin dispersion within the matrix. These agglomerates typically act as stress concentration sites, thereby reducing the mechanical performance of the composites due to weak interfacial bonding. In contrast, the samples containing 0.25 wt% lignin and MA exhibited a more uniform morphology, with smooth fracture surfaces free of cracks or particle clusters. This suggests improved interfacial compatibility and stress transfer efficiency, which are expected to enhance the material’s mechanical properties.

At higher lignin contents, lignin nanoparticles tended to agglomerate because of their high surface energy and strong intermolecular interactions. These lignin agglomerates act as stress concentration sites and reduce the effective interfacial contact area between the lignin particles and the PLA matrix. Consequently, stress transfer from the polymer matrix to the reinforcement becomes less efficient, promoting premature crack initiation and propagation under mechanical loading. In addition, lignin agglomeration results in a non-uniform particle distribution, limiting the reinforcing effect of lignin and reducing tensile and flexural properties. The SEM observations in this study support this mechanism, as larger agglomerates were observed in composites with higher lignin loadings.

### 3.3. Prediction Results

PLA, lignin, and MA were determined as input values. The output values were determined as flexural strength, flexural modulus, tensile strength, tensile modulus, and EatB. A total of 80% of these data were used for training, and the remaining 20% for testing. R^2^, RMSE, and MAE were calculated by comparing predicted values with actual values. By comparing the R^2^ values, the effectiveness of the ML models, including RF and XGBoost, was evaluated. The specific performance of every ML model is displayed in Table 3. The best results are bolded for emphasis.

When the results in the table are examined, both machine learning models show quite high performance. Based on the evaluation of R^2^, RMSE, and MAE, the Random Forest (RF) model yields better results than the XGBoost model. While the RF model explains the dependent variable better, with an R^2^ of 0.9979, it also surpasses the XGBoost model in RMSE and MAE, with values of 0.0158 and 0.0120, respectively. The XGBoost model achieves high accuracy (R^2^ = 0.9950) but has slightly higher error than the RF model.

Although both models achieved very high prediction accuracy, the relatively small experimental dataset should be considered when interpreting these results. The reported R^2^ values were obtained from an independent test set generated using a single random train–test split. Therefore, the models should primarily be regarded as interpolation models within the investigated composition range. While the consistently low RMSE and MAE values suggest good predictive consistency, additional validation using larger datasets, repeated random splits, or k-fold cross-validation would provide a more rigorous assessment of model generalization and further reduce the possibility of overfitting.

**Figure 7:**
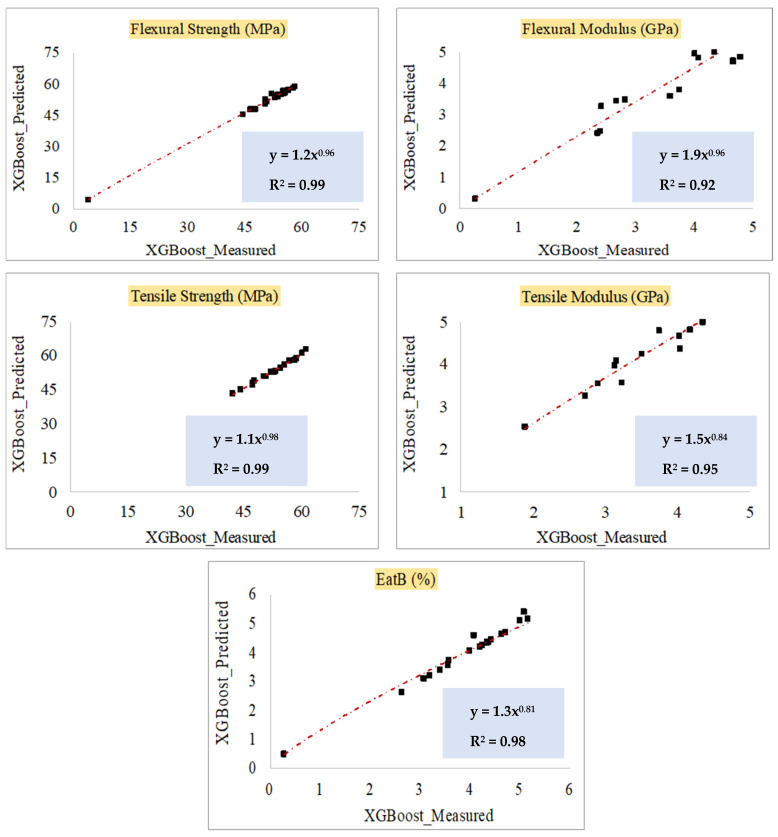
R^2^ values of estimated (XGBoost) and measured bending properties.

The five graphs in Figure 7 show the strength of the linear relationship between the XGBoost-predicted values and the measured values. In the flexural strength (MPa) graph, a very strong relationship between the predicted and measured values was observed, with an R^2^ value of 0.995. Similarly, as seen in the tensile strength (MPa) graph, the model made very successful predictions, with an R^2^ value of 0.9891. The flexural modulus (GPa) and tensile modulus (GPa) graphs also showed high accuracy, with R^2^ values of 0.9235 and 0.9449, respectively. These high R^2^ values, especially observed in the modulus values, show that the model was also successful in predicting elastic properties. Finally, in the EatB (%) graph, the R^2^ value for the relationship between the predicted and measured values is 0.9764, indicating that the model also accurately predicted the post-bending fracture elongation parameter.

**Figure 8:**
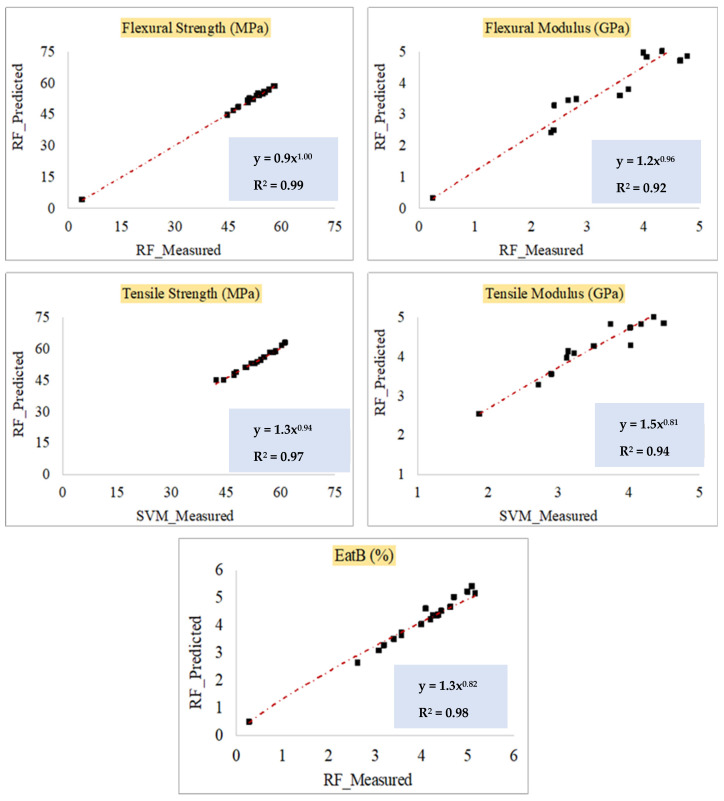
R^2^ values of estimated (RF) and measured bending properties.

**Figure 9:**
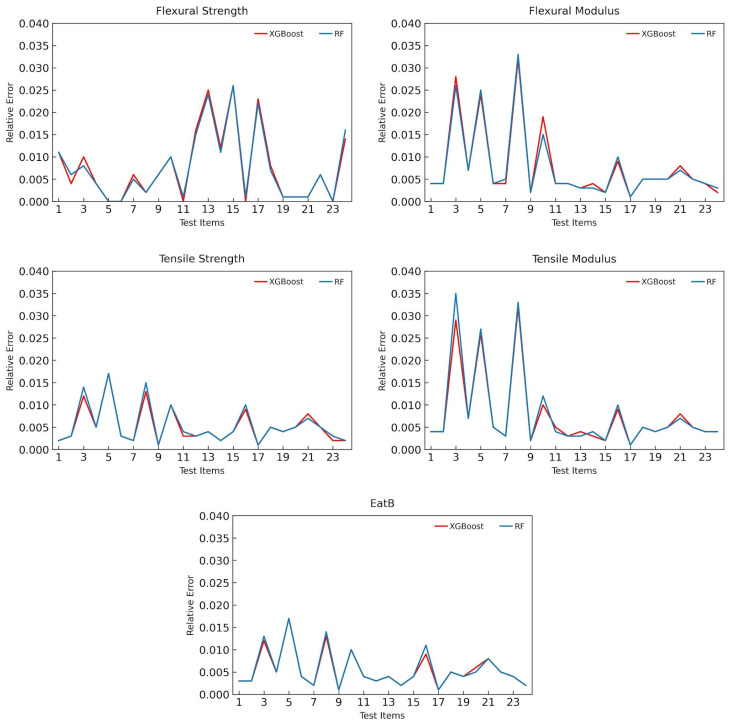
Relative error for XGBoost and RF.

The five different graphs in Figure 8 show the relationship between the values predicted by the Random Forest (RF) model and the experimentally measured values. In the flexural strength (MPa) graph, the linear relationship between the predicted and measured values is strong, and the model performs well, with an R^2^ value of 0.9979. Although the model’s performance for tensile strength (MPa) is slightly lower, it is still quite strong, with an R^2^ value of 0.9794. In the flexural modulus (GPa) and tensile modulus (GPa) graphs representing elastic properties, the R^2^ values are 0.9208 and 0.9359, respectively. These values indicate that the RF model is reasonably successful at predicting moduli. Finally, in the EatB (%) graph, the R^2^ value is 0.9757, indicating that the model exhibits very strong predictive performance for this parameter. The relative errors for XGBoost and RF are shown in Figure 9.

The five graphs in Figure 9 show the relative error rates for values estimated by the XGBoost and Random Forest (RF) models. In all graphs, the test numbers are on the horizontal axis, and the relative errors (%) are on the vertical axis. As shown in the flexural strength (MPa) and tensile strength (MPa) graphs, both models produced very low error rates, and the relative error values generally ranged between 0.005 and 0.020. It is particularly striking that the XGBoost model produced more balanced, lower error values than RF in the tensile strength graph. In the flexural modulus (GPa) and tensile modulus (GPa) graphs, it can be observed that the error rates reach up to 0.030 in some test samples, but these deviations show a similar trend for both models. This situation shows that modulus estimates are a bit more challenging than strength parameters. In the EatB (%) graph, it is seen that the error values of both models are evenly distributed between 0.005 and 0.020, and the error curve of the XGBoost model is more stable than that of RF.

## 4. Conclusions

In this study, machine learning-assisted optimization of the mechanical and morphological properties of PLA-based nanocomposites reinforced with lignin nanoparticles and maleic anhydride (MA) was conducted. Lignin, obtained through an environmentally friendly method, was incorporated into the PLA matrix together with various amounts of MA and processed using a co-rotating twin-screw extrusion system, followed by injection molding. Mechanical testing revealed that low lignin content (≤1 wt%) combined with moderate MA content (3–5 wt%) positively influenced both mechanical strength and ductility. Notably, the PLA-5MA-5L formulation exhibited the highest elongation at break (EatB) value of 5.1%, while PLA-3MA-0.5L showed the highest flexural strength of 58.1 MPa. SEM analyses demonstrated more homogeneous dispersion and smooth fracture surfaces in samples with low lignin content. This indicates morphological deterioration, as higher lignin loadings resulted in particle agglomeration, void formation, and crack development. These findings show that high lignin content limited mechanical performance and played a critical role in matrix–filler interactions.

Prediction results obtained with machine learning algorithms, particularly the Random Forest (RF) model, showed excellent agreement with the experimental data, with high R^2^ values and low error metrics. This implies that composite formulations can be optimized with fewer experimental trials, contributing to time and resource efficiency. Although feature importance analysis was beyond the scope of the present study, future work will investigate the relative contributions of lignin and MA contents using SHAP values or impurity-based feature importance to improve model interpretability. The developed models should only be applied within the investigated composition ranges (0–5 wt.% lignin and 0–10 wt.% MA). Predictions beyond these limits represent extrapolation and should therefore be interpreted with caution until validated experimentally. In conclusion, it can be said that the development of biodegradable, eco-friendly, and mechanically balanced PLA-based nanocomposites requires careful adjustment of lignin and MA content, and that data-driven approaches, such as ML-based modeling, can serve as powerful tools in this process. Therefore, the findings constitute a valuable reference for future research on the digitalization of material design and production processes. The proposed models are most reliable within the investigated composition range and should be used cautiously for extrapolation beyond this domain. In addition to the experimental findings, integrating machine learning provided a powerful framework for understanding and optimizing the composition–property relationships of PLA-based nanocomposites. The high predictive performance of both models, particularly the Random Forest algorithm, demonstrates that reliable predictions can be achieved even with relatively small experimental datasets. This highlights the robustness of ensemble learning approaches in capturing nonlinear interactions between lignin and MA contents. Furthermore, the ML-assisted approach significantly reduces the need for extensive experimental trials, offering a time- and cost-efficient strategy for material design. Therefore, the proposed methodology not only enhances the current study but also provides a scalable, transferable approach for future research on biopolymer composites and other multicomponent material systems.

## Figures and Tables

**Table 1:** Experimental design.

Samples	PLA	Lignin	MA
Neat PLA	100		
PLA-0.25%L	99.75	0.25	
PLA-0.5%L	99.5	0.5	
PLA-1%L	99	1	
PLA-5%L	95	5	
PLA-3MA	97		3
PLA-3MA-0.25%L	96.75	0.25	3
PLA-3MA-0.5%L	96.5	0.5	3
PLA-3MA-1%L	96	1	3
PLA-3MA-%5L	92	5	3
PLA-5MA	95		5
PLA-5MA-0.25%L	94.75	0.25	5
PLA-5MA-0.5%L	94.5	0.5	5
PLA-5MA-1%L	94	1	5
PLA-5MA-%5L	90	5	5
PLA-10MA	90		10
PLA-10MA-0.25%L	89.75	0.25	10
PLA-10MA-0.5%L	89.5	0.5	10
PLA-10MA-1%L	89	1	10
PLA-10MA-%5L	85	5	10

**Table 2:** Mechanical properties of the lignin-reinforced PLA composites.

Samples	TS		TM		FS		FM		EatB	
	s (±)	DT	s (±)	DT	s (±)	DT	s (±)	DT	s (±)	DT
**Neat PLA**	1.7	A	0.1	E	0.5	EFGH	0.05	A	0.1	DEFG
**PLA-0.25%L**	2.1	BCD	0.2	G	1.4	AB	0.1	A	0.4	EFGH
**PLA-0.5%L**	1.1	CDEF	0.2	F	1	BCD	0.2	A	0.2	ABC
**PLA-1%L**	2.6	BCD	0.1	E	1.6	DEFG	0.3	B	0.3	ABC
**PLA-5%L**	2.1	A	0.2	E	1.5	CDE	0.1	BCDE	0.1	ABC
**PLA-3%MA**	1.2	DEF	0.1	EF	0.9	BCD	0.03	BCDE	0.3	EFGHI
**PLA-3%MA-0.25%L**	0.5	EF	0.1	F	0.5	DEFG	0.08	DE	0.5	BCDE
**PLA-3%MA-0.5%L**	1	BCDE	0.3	F	1	DEFGH	0.1	DE	0.4	FGHI
**PLA-3%MA-1%L**	2.1	BC	0.1	D	1	H	0.09	BCDE	0.2	GHI
**PLA-3%MA-5%L**	2.7	AB	0.2	D	0.5	DEFGH	0.07	DE	0.3	EFGHI
**PLA-5%MA**	1	CDEF	0.1	EF	1	FGH	0.06	BC	0.2	EFGH
**PLA-5%MA-0.25%L**	1.7	ABC	0.2	EF	1	GH	0.06	CDE	0.2	EFGHI
**PLA-5%MA-0.5%L**	1.2	CDEF	0.2	D	4	BCD	0.1	E	0.3	ABCD
**PLA-5%MA-1%L**	1.1	BCDE	0.1	C	3	AB	0.1	DE	0.4	DEF
**PLA-5%MA-5%L**	0.5	A	0.1	A	2	DEFGH	0.07	BCDE	0.6	HI
**PLA-10%MA**	1	AB	0.1	B	0.5	FGH	0.03	A	0.4	FGHI
**PLA-10%MA-0.25%L**	0.3	ABC	0.1	B	0.8	DEFGH	0.1	DE	0.2	BCDE
**PLA-10%MA-0.5%L**	3.5	ABC	0.2	B	0.9	CDEF	0.09	A	0.3	I
**PLA-10%MA-1%L**	1.5	AB	0.2	B	0.7	EFGH	0.07	A	0.1	CDEF
**PLA-10%MA-5%L**	2	A	0.2	B	0.9	ABC	0.08	BCDE	0.2	EFGH

**Table 3:** Prediction values obtained with XGBoost and RF.

ML Models	R^2^	RMSE	MAE
XGBoost	0.9950	0.0160	0.0121
RF	0.9979	0.0158	0.0120

## Data Availability

The original contributions presented in the study are included in the article; further inquiries can be directed to the corresponding author.

## Abbreviations

The following abbreviations are used in this manuscript:

NLPs	Lignin nanoparticles
MA	Maleic anhydride
PLA	Polylactic acid
SEM	Scanning electron microscopy
RF	Random Forest
XGBoost	Extreme Gradient Boosting
PHB	Polyhydroxybutyrate
DES	Deep eutectic solvent
PEG	Poly(ethylene glycol)
ML	Machine learning
MLP	Multilayer perceptron
GBDT	Gradient Boosted Decision Tree
MSE	Mean squared error
DT	Decision tree
MAE	Mean Absolute Error
RMSE	Root Mean Square Error
TS	Tensile strength
TM	Tensile modulus
FS	Flexural strength
FM	Flexural modulus
